# Wnt-dependent mechanism of the apical constriction of roof plate cells in developing mouse spinal cord

**DOI:** 10.3389/fcell.2025.1571770

**Published:** 2025-07-07

**Authors:** Takuma Shinozuka, Tadashi Okubo, Noriaki Sasai, Shinji Takada

**Affiliations:** ^1^ Exploratory Research Center on Life and Living Systems, National Institutes of Natural Sciences, Okazaki, Aichi, Japan; ^2^ National Institute for Basic Biology, National Institutes of Natural Sciences, Okazaki, Aichi, Japan; ^3^ Division of Biological Science, Nara Institute of Science and Technology, Ikoma, Nara, Japan; ^4^ Department of Laboratory Animal Science, Kitasato University School of Medicine, Sagamihara, Kanagawa, Japan; ^5^ Basic Biology Program, The Graduate University for Advanced Studies (SOKENDAI), Okazaki, Aichi, Japan

**Keywords:** Wnt, roof plate, neural tube, apical constriction, morphogenesis, cytoskeleton

## Abstract

Apical constriction of epithelial cells usually occurs in a local portion of epithelial sheet, which results in bending of epithelial tissues. However, it is uncertain whether diffusible signal molecules, like Wnt, regulate such locally restricted events. Here, we show that Wnt ligands are required for apical constriction of Wnt1-expressing roof plate (RP) cells during development of the neural tube. Analysis of *Wntless* conditional knock-out (cKO) embryos, in which Wnt secretion from Wnt1-expressing roof plate cells is impaired, revealed that RP-derived Wnt ligands are required for phosphorylation of myosin light chain (MLC) and apical constriction of RP cells. Loss- or gain-of-function analysis of β-catenin reveals that this apical constriction is regulated in a β-catenin-dependent manner. Consistent with the timing of apical constriction, Wnt ligands accumulate on the apical side of RP cells. In embryos with Wnt1-expressing RP-specific defects in synthesis of heparan sulfate proteoglycan, apical accumulation of Wnt ligands and apical constriction are impaired. Therefore, we propose that specific accumulation of Wnt ligands on RP cells drives apical constriction of these cells.

## Introduction

During development, epithelial cells often change their morphology. Morphological transformation of epithelial cells appears to be highly regulated in many stages of tissue morphogenesis. Apical constriction is one of the morphogenetic processes of epithelial cells. During apical constriction, the length of the apical surface of epithelial cells is reduced, giving cells a wedge shape (Martin and Goldstein, 2014). In many cases, apical constriction occurs in a localized area, and such regionally restricted cell shape changes transform the epithelial sheet in embryonic morphogenesis, during gastrulation, neurulation, and neural tube formation (Leptin and Grunewald, 1990; Haigo et al., 2003; Sawyer et al., 2010; Nikolopoulou et al., 2017). Apical constriction is induced by contraction of the actomyosin cytoskeleton that localizes and binds to apical cell membranes (Martin and Goldstein, 2014; Hunter and Fernandez-Gonzalez, 2017). This contraction is regulated by phosphorylation of myosin light chain (MLC), which is induced by kinases such as ROCK and MLCK (Kinoshita et al., 2008; Vicente-Manzanares et al., 2009; Escuin et al., 2015). To understand tissue morphogenesis, it is important to identify regulatory mechanisms of apical constriction of developmental epithelial cells in each context.

The neural tube is a typical epithelial structure generated in early gestation. In developing neural tube, the roof plate (RP) serves as an organizing center along the dorsal midline (Chizhikov and Millen, 2004). During neural development, RP cells produce secreted signaling molecules, such as Wnt and BMP. These signaling molecules regulate cell proliferation and specification of neuroepithelial cells in the dorsal spinal cord, as well as neural crest cells, which originate from the RP (Ikeya et al., 1997; Lee and Jessell, 1999; Muroyama et al., 2002; Wine-Lee et al., 2004). After the initial events regulated by RP-derived signals, morphology of the spinal cord changes dynamically. The spinal cord lumen becomes a flattened cavity with a short left-right axis, followed by gradual shrinkage along the dorsoventral (D-V) axis (Sturrock, 1981; Sevc et al., 2009; Canizares et al., 2020). In the course of this shrinkage, dorsal neuroepithelial cells, with the exception of RP cells, gradually lose their apical surface contact with the lumen. This is called dorsal collapse and the loss of contact occurs in a dorsal to ventral direction (Canizares et al., 2020; Tait et al., 2020). Upon dorsal collapse, RP cells are stretched along the D-V axis and elongated processes of RP cells align along the future dorsal septum of the spinal cord while maintaining contact with the most dorsal portion of the shrinking lumen, which finally develops into the central canal of the spinal cord. At this contact site, the apical surface of each RP cell is highly contracted and many RP cells are densely assembled (Shinozuka et al., 2019). This contraction results in dense organization of cell projections, which probably increases the physical strength of the bundle of RP cells in the septum of the spinal cord (Korzh, 2014; Shinozuka and Takada, 2021). Thus, this contraction appears to be important for proper transformation of RP cells, as well as the neural tube.

In mouse and other vertebrate embryos, Wnt1 and Wnt3a, which mainly activate Wnt/β-catenin signaling, are specifically produced in the RP (Clevers, 2006; Parr et al., 1993). These Wnt ligands promote proliferation and specification of dorsal neuroepithelial cells and neural crest cells (Ikeya et al., 1997; Muroyama et al., 2002). In the dorsal neural tube, Wnt signaling, which is detectable by expression of Wnt target genes such as *Axin2*, is widely activated in a gradient along the D-V axis (Jho et al., 2002). On the other hand, we recently found that RP-derived Wnt ligands are also required for stretching morphogenesis of RP cells (Shinozuka et al., 2019). In RP, specific, conditional knock-out embryos for *Wntless* (*Wls* cKO), which is required for Wnt secretion (Goodman et al., 2006; Bartscherer et al., 2006; Banziger et al., 2006), morphology of elongated RP cells is abnormal. Moreover, electron microscopic analysis reveals that their apical surface is widened in *Wls* cKO RP cells at E18.5 (Shinozuka et al., 2019). Thus, in addition to proliferation and specification of dorsal neural cells, Wnt signaling is required for the morphological change of RP cells, including shrinkage of apical surfaces. However, it remains unclear when and how Wnt signal regulates morphological transformation of RP cells.

To better understand this issue, we followed RP cells in mouse embryos after neural tube closure. We found that phosphorylation of myosin light chain (MLC) was specifically increased in RP cells, coincident with morphological transformation of these cells. Analyses of a series of mutant embryos impaired in Wnt signaling revealed that MLC phosphorylation is promoted by RP-derived Wnt ligands via the Wnt/β-catenin pathway. Coincident with this phosphorylation, Wnt proteins accumulate specifically on apical surfaces of RP cells and embryos defective in this Wnt accumulation manifest impaired apical constriction of RP cells. Thus, we propose that diffusible Wnt ligands also act locally in neural development.

## Results

### Apical constriction occurs in the roof plate region

**FIGURE 1:**
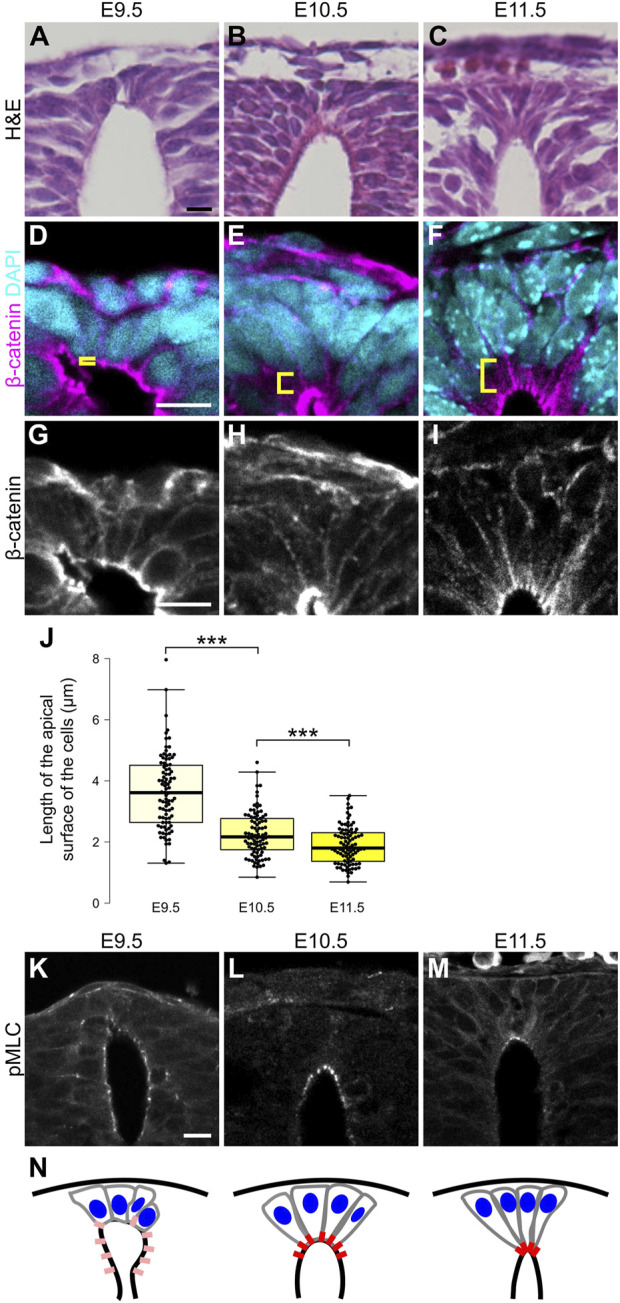
Apical constriction occurs in roof plate. **(A–C)** Hematoxylin and eosin (H&E) staining of the transverse sections at E9.5 **(A)**, E10.5 **(B)** and E11.5 **(C)**. **(D–M)** Transverse sections at the forelimb level showing apical constriction of RP cells at E9.5 **(D,G,K)**, E10.5 **(E,H,L)** and E11.5 **(F,I,M)** using anti-β-catenin **(D–I)** and anti-pMLC **(K–M)** antibodies. A statistical summary of the length of the apical surface of RP cells is shown in **(J)**. The length of the apical surface of RP cells (the 10 most dorsal cells per section) was quantified based on localization of beta-catenin at the apical membrane. For each developmental stage, a total of 90 cells from three embryos were examined. ***P < 0.001 (Student’s *t*-test). Nuclei were counterstained with DAPI. Brackets indicate the region from the apical surface to the nucleus of each cell. Three embryos were examined in each experiment. **(N)** Schematic of RP cell morphology and distribution of pMLC based on K-M. Scale bars: 10 µm.

During neural development, RP cells change their morphology, and develop a wedge-like form. To determine the timing of morphological transformation of RP cells, we analyzed shapes of RP cells from E9.5 to E11.5. At E9.5, RP cells exhibit a round shape with broad apical surfaces (Figures 1A,D,G,J). After E10.5. Their apical surfaces shrink, and their wedge-like form is maintained thereafter (Figures 1B,C,E,F,H–J). This morphological change occurs simultaneously with repositioning of nuclei from apical to basal in RP cells (Figures 1D–F). Immunohistochemical staining for phosphorylation of myosin light chain (MLC) confirmed that it causes contraction of the actomyosin cytoskeleton. While phosphorylation of MLC was widely detected in the lumen of the spinal cord at E9.5, its signal became restricted to apical surfaces of RP cells after E10.5 (Figures 1K–M). These findings suggest that actin mediated constriction occurs on the apical side of RP at E10.5 (Figure 1N).

### Wnt secretion from roof plate cells is required for apical constriction

**FIGURE 2:**
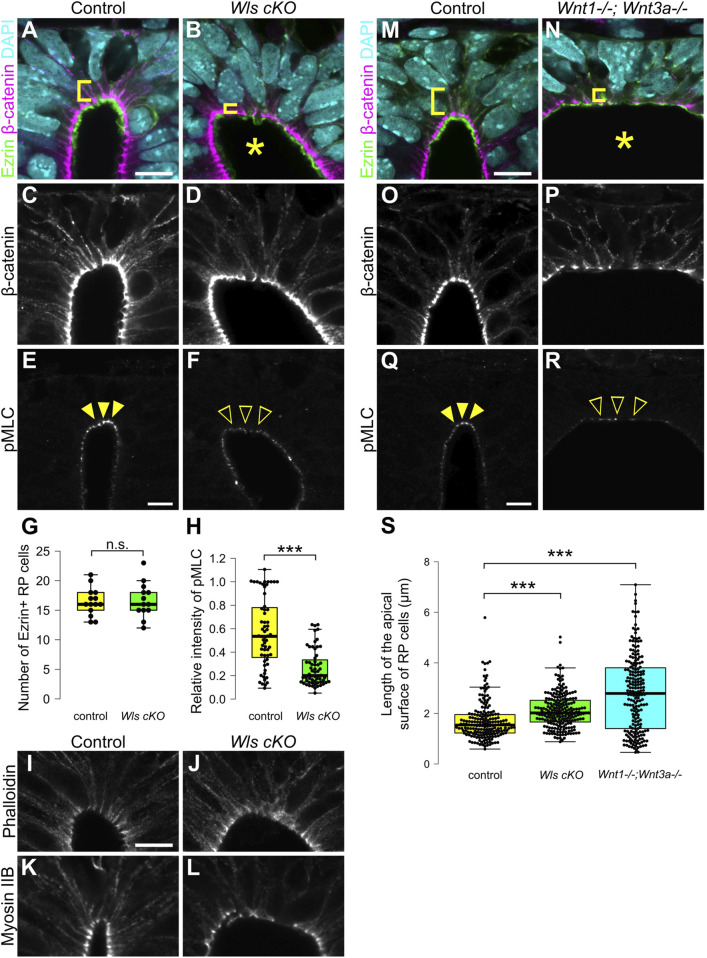
Wnt secretion from roof plate cells is required for apical constriction. **(A–L)** Transverse sections at the forelimb level in *Wls* cKO embryos **(B,D,F,J,L)** and littermate controls **(A,C,E,I,K)** at E11.5. To visualize morphology of RP cells, we stained sections with anti-Ezrin **(A,B)** and anti-β-catenin antibodies **(A–D)**. To visualize the actomyosin cytoskeleton, we stained sections with anti-pMLC **(E,F)** antibody, phalloidin **(I,J)** and anti-Myosin IIB **(K,L)** antibody. A statistical summary indicating the number of Ezrin-positive RP cells is shown in **(G)**. A total of 14 sections from four control and 13 sections from four *Wls* cKO embryos were examined. n.s., not significant. A statistical summary of the relative intensity of pMLC normalized to β-catenin signal intensity is shown in **(H)**. For each genotype, a total of 60 cells from three embryos were examined. ***P < 0.001 (Student’s *t*-test). **(M–S)** Transverse sections at the forelimb level in *Wnt1; Wnt3a* double KO embryos **(N,P,R)** and littermate controls **(M,O,Q)** at E11.5 stained with anti-Ezrin **(M,N)**, anti-β-catenin **(M–P)** and anti-pMLC **(Q,R)** antibodies. Nuclei were counterstained with DAPI. An asterisk indicates expansion of the apical surface. Arrowheads indicate pMLC signals in RP cells in control, while open arrowheads indicate reduced signals in *Wls* cKO or *Wnt1; Wnt3a* double KO embryos. Three embryos were examined in each experiment. A statistical summary indicating the length of the apical surface of RP cells is shown in **(S)**. The length of the apical surface of Ezrin-positive roof plate cells was quantified based on the localization of beta-catenin at the apical membrane. A total of 192 cells prepared from three control, 218 cells from four *Wls* cKO and 204 cells from three *Wnt1; Wnt3a* double KO embryos were examined. ***P < 0.001 (Student’s *t*-test). Scale bars: 10 µm.

Previously, analysis of *Wntless* conditional knock-out embryos (*Wls* cKO), in which Wnt secretion is specifically defective in Wnt1-expressing RP cells, revealed impaired patterning of dorsal neural progenitor cells without affecting RP cell specification. Of note, it also revealed that Wnt signaling from RP cells is required for morphological transformation of RP cells, including apical surface expansion during late developmental stages, and cell proliferation around the dorsal central canal (Shinozuka et al., 2019). To reveal whether apical constriction of RP cells is regulated by Wnt signaling, we performed quantitative analysis of RP cell morphology in *Wls* cKO embryos (Carpenter et al., 2010; Danielian et al., 1998). In *Wls* cKO embryos, the length of the apical surface of RP cells was significantly expanded compared with control embryos at E11.5 (Figures 2A–D,S). The number of Ezrin-positive RP cells was unchanged between control and *Wls* cKO embryos, suggesting that apical surface expansion is not due to altered RP cell numbers (Figure 2G). These data indicate that Wnt ligands, secreted from RP cells, regulate constriction of the apical surface of these cells, showing that Wnt signaling is required for the morphological change of RP cells during early stages of neural development.

To identify regulatory mechanisms of the morphological change of RP cells, we analyzed the actomyosin cytoskeleton. Contraction of actomyosin cytoskeleton caused by phosphorylation of MLC drives apical constriction (Komatsu et al., 2000; Martin and Goldstein, 2014). First, we analyzed phosphorylation of MLC. In control embryos, phosphorylation of MLC is restricted to apical surfaces of RP cells (Figure 2E). In contrast, phosphorylation of MLC is significantly reduced in *Wls* cKO embryos (Figures 2F,H). Next, we analyzed the distribution of the actomyosin cytoskeleton in *Wls* cKO embryos. At E11.5, actin and myosin are found at the apical surface of the spinal cord (Figures 2I,K). In *Wls* cKO embryos, localization of the actomyosin cytoskeleton did not change compared with control embryos (Figures 2J,L). These data indicate that Wnt signaling regulates phosphorylation of MLC, but not localization of the actomyosin cytoskeleton and suggests that Wnt signaling is required for apical constriction of RP cells.

### *Wnt1* and *Wnt3a* are required for apical constriction

Next, we analyzed which ligands are involved in apical constriction and phosphorylation of MLC. In RP cells, *Wnt1* and *Wnt3a* are specifically expressed at the time of apical constriction (McMahon et al., 1992; Parr et al., 1993; Wilkinson et al., 1987). To investigate whether Wnt1 and Wnt3a are required for apical constriction of RP cells, we generated *Wnt1* and *Wnt3a* double KO embryos, which have previously been shown not to affect RP cell specification (McMahon and Bradley, 1990; Takada et al., 1994; Muroyama et al., 2002). The apical surface of RP cells significantly expanded in *Wnt1* and *Wnt3a* double-KO embryos at E11.5 (Figures 2M–P,S). Moreover, phosphorylation of MLC was significantly reduced in *Wnt1* and *Wnt3a* double-KO embryos (Figures 2Q,R). The dorsal lumen appeared more expanded than in *Wls* cKO embryos (Figures 2A–D,M–P). Since a similar phenotype is observed in Wnt3a KO embryos, this expansion of the dorsal lumen is likely caused by the physical effects of Wnt3a deficiency, resulting in opening of the posterior trunk neural tube (Takada et al., 1994). These data suggest that activation of the actomyosin cytoskeleton is regulated by Wnt1 and Wnt3a secreted from RP cells.

### Apical constriction of roof plate cells occurs in a *β-catenin*-dependent manner

**FIGURE 3:**
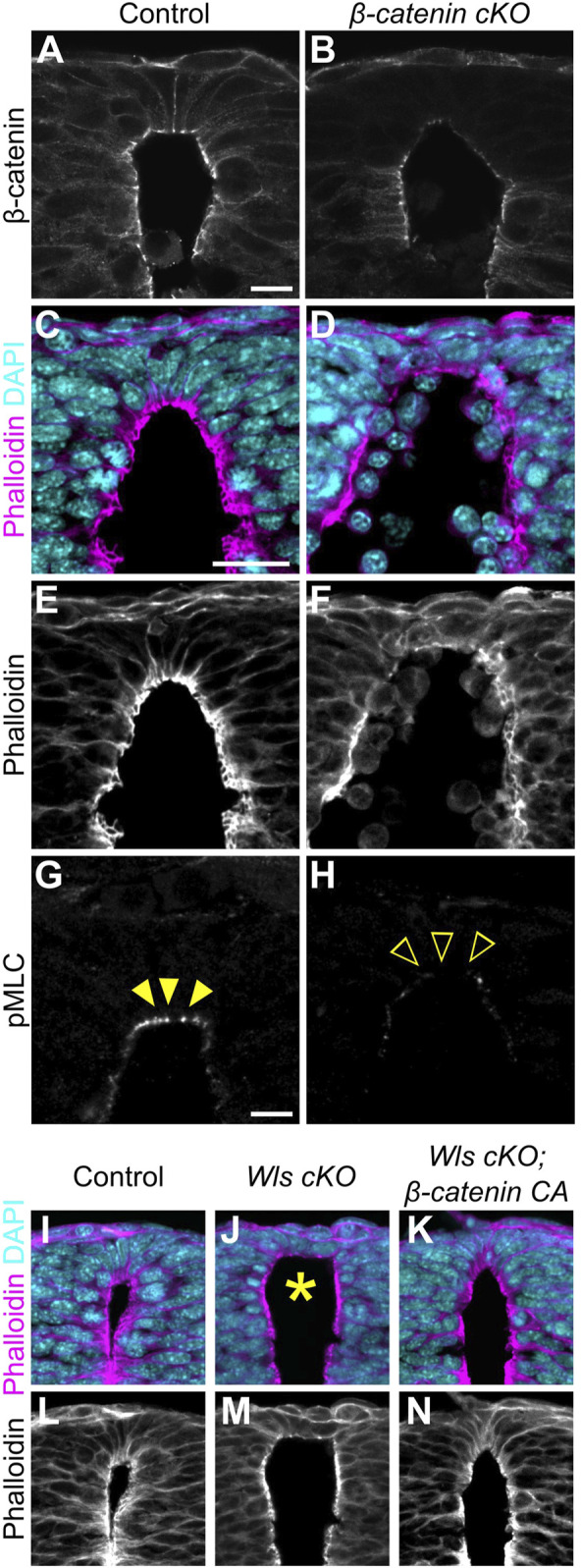
Apical constriction in roof plate cells occurs in a β-catenin-dependent manner. **(A–H)** Transverse sections at the forelimb level in *β-catenin* cKO embryos **(B,D,F,H)** and littermate controls **(A,C,E,G)** at E10.5 stained with anti-β-catenin **(A,B)** antibody, phalloidin **(C–F)** and anti-pMLC **(G,H)** antibody. **(I–N)** Transverse sections at the forelimb level in control **(I,L)**, *Wls* cKO **(J,M)** and *Wls* cKO; *β-catenin* CA **(K,N)** embryos at E10.5 stained with phalloidin. Nuclei were counterstained with DAPI. Arrowheads indicate pMLC signals in RP cells, while open arrowheads indicate reduced signals. An asterisk indicates expansion at the apical surface. Three embryos were examined in each experiment. Scale bars: 10 µm.

In general, *Wnt1* and *Wnt3a* are thought to induce the canonical Wnt/β-catenin pathway (Niehrs, 2012). To reveal whether apical constriction in RP is β-catenin-dependent, we generated RP-specific *β-catenin*-cKO-embryos by crossing of floxed *β-catenin* mice with those carrying the *Wnt1-Cre* transgene (Danielian et al., 1998; Brault et al., 2001). We confirmed that in *β-catenin* cKO embryos, β-catenin is significantly reduced in the RP region (Figures 3A,B). Consistent with *Wls* cKO or *Wnt1-*and-*Wnt3a*-double-KO embryos, *β-catenin*-cKO embryos showed expansion of apical surfaces and disrupted nuclear positioning (Figures 3C–F). Moreover, phosphorylation of MLC is significantly reduced in RP cells (Figures 3G,H), suggesting that apical constriction in RP is induced in a β-catenin-dependent manner.

Then, to examine whether apical constriction is induced by Wnt signals in a β-catenin-dependent manner, we performed a rescue experiment in *Wls* cKO embryos employing β-catenin gain-of-function. We induced RP-specific activation of β-catenin by crossing exon3 floxed *β-catenin* mice with those carrying the *Wnt1-Cre* transgene (Danielian et al., 1998; Harada et al., 1999). Deletion of β-catenin exon3 stabilized β-catenin in a constitutive active form (Iwao et al., 1998; Harada et al., 1999). While *Wls*-cKO embryos showed expanded apical surfaces and disrupted nuclear positioning, β-catenin gain-of-function in *Wls*-cKO embryos showed no obvious abnormality in apical cell surface morphology or apico-basal position of nuclei (Figures 3I–N). These findings suggest that apical constriction in RP is regulated primarily by canonical Wnt/β-catenin signaling. However, since β-catenin also functions in cell adhesion, changes in cell adhesion strength may also influence this phenotype.

### HSPGs produced by roof plate cells are required for apical constriction

**FIGURE 4:**
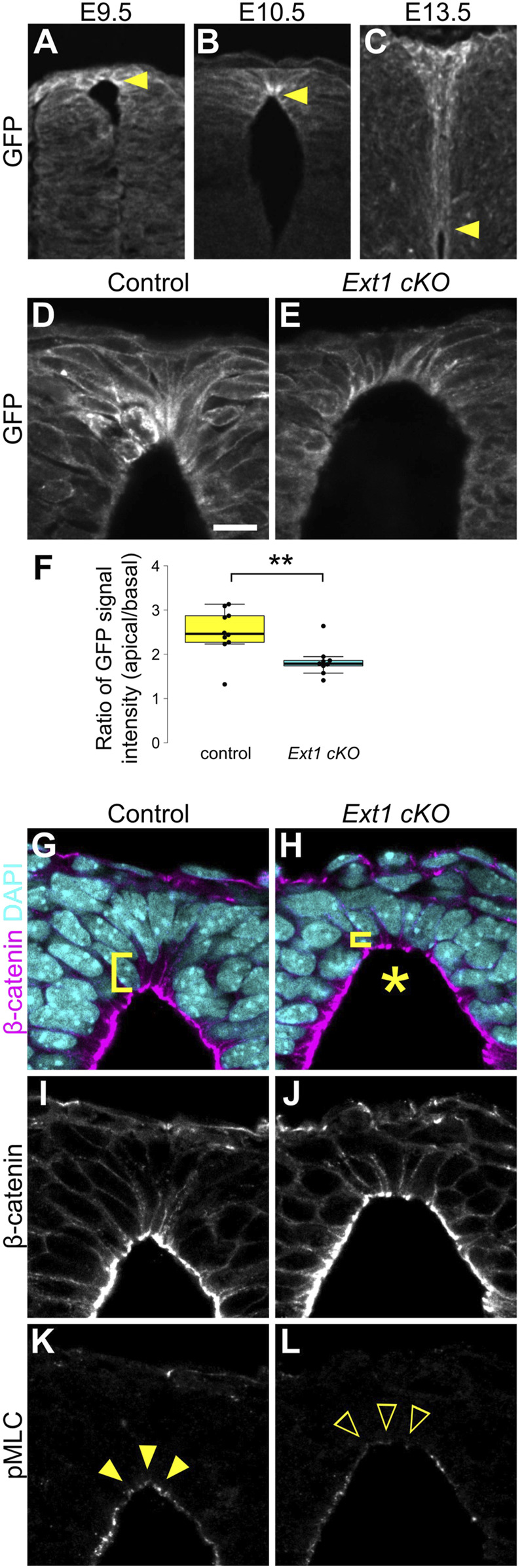
HSPGs are required for apical constriction. **(A–C)** Transverse sections of *EGFP-Wnt3a* knock-in embryos at the forelimb level in the developing spinal cord at E9.5 **(A)**, E10.5 **(B)** and E13.5 **(C)** were stained with anti-GFP antibody. Arrowheads indicate the apical tips of RP cells. **(D–L)** Transverse sections at the forelimb level in *Ext1*-cKO embryos **(E,H,J,L)** and littermate controls **(D,G,I,K)** at E10.5 using anti-GFP **(D,E)**, anti-β-catenin **(G–J)** and anti-pMLC **(K,L)** antibodies. Nuclei were counterstained with DAPI. Brackets indicate the region from the apical surface to the nucleus of each cell. An asterisk indicates expansion of the apical surface. Arrowheads indicate pMLC signals in RP cells, while open arrowheads indicate reduced signals. A statistical summary indicating the ratio of the GFP signal intensity from apical to basal is shown in **(F)**. A total of 9 sections from two *Ext1* cKO and 10 sections from two littermate control embryos were examined. **P < 0.01 (Student’s *t*-test). Scale bar: 10 µm.

Although Wnt/β-catenin signaling is widely activated in dorsal neural tube, apical constriction was observed only in RP cells. To understand why this discrepancy occurs, we examined Wnt protein distribution around RP cells using *EGFP-Wnt3a* knock-in mice, whose distribution pattern is consistent with that of endogenous Wnt3a (Shinozuka et al., 2019). At E9.5, Wnt3a proteins are uniformly detected in RP cells. Interestingly, Wnt proteins accumulate specifically at apical surfaces of RP cells at E10.5, coincident with the wedge-shaped morphological change (Figures 4A,B). Accumulation of Wnt3a proteins on apical surfaces of RP cells decreases at E13.5, when RP cells begin to elongate along the D-V axis (Figure 4C). This suggests that temporal accumulation of Wnt proteins is correlated with apical constriction of RP cells.

Heparan sulfate proteoglycans (HSPGs) bind secretory signaling molecules, including Wnt, and control their spatial distribution and signaling activity (Baeg et al., 2001; Han et al., 2005; Mii et al., 2017; Suzuki et al., 2024). Therefore, we speculated that HSPGs are involved in accumulation of Wnt ligands in RP cells. To address this, we generated RP-specific *Ext1* cKO mice by crossing floxed *Ext1* mice with those carrying the *Wnt1-Cre* transgene (Danielian et al., 1998). Ext1 is an extending enzyme of heparan sulfate chains. In contrast to normal embryos, accumulation of Wnt3a proteins was significantly reduced in *Ext1*-cKO embryos (Figures 4D–F). Furthermore, consistent with the phenotype of Wnt mutant embryos, expansion of apical surfaces and disrupted nuclear positioning were observed in *Ext1*-cKO embryos (Figures 4G–J). Moreover, phosphorylation of MLC is significantly reduced in RP cells (Figures 4K,L), suggesting that HSPGs regulate Wnt protein distribution and are required for local activation of Wnt signaling in RP at the time of apical constriction.

## Discussion

In summary, this study revealed that Wnt/β-catenin signaling is required and sufficient for myosin activation and apical constriction in RP cells in neural tube development. Consistent with these changes, Wnt proteins secreted by RP cells accumulate at apical surfaces of RP cells, depending on HSPG. Thus, we propose that this accumulation of Wnt ligands triggers morphological changes of RP cells via Wnt/β-catenin signaling-mediating modulation of the actomyosin cytoskeleton.

Secreted signaling molecules, including Wnt, are thought to act as morphogens that diffuse globally from producing cells, forming concentration gradients (Zecca et al., 1996; Neumann and Cohen, 1997). In contrast, it has also been reported that Wnt proteins accumulate locally and act on cells close to producing cells (Alexandre et al., 2014; Farin et al., 2016; Shinozuka et al., 2019; Hatakeyama et al., 2023). For instance, locally applied Wnt ligands induce asymmetric distribution of Wnt–β-catenin signaling components and orient asymmetric cell division of ES cells (Habib et al., 2013). In the developing neural tube, Wnt signaling, detectable by expression of the Wnt target gene, *Axin2*, is activated in a gradient along the D-V axis (Jho et al., 2002). However, this study revealed that Wnt proteins also accumulate locally at the apical side of RP cells, at least at a particular stage of neural tube development and regulate apical constriction in RP cells. Notably, such Wnt accumulation was not observed in other regions undergoing apical constriction, such as the dorsolateral hinges. Thus, we propose that Wnt proteins secreted from RP cells regulate neural tube development by both global and local actions.

Our results also suggest that HSPGs trap Wnt ligands and restrict the range of highly activated Wnt signaling to a small number of cells. HSPGs regulate distribution of secreted signaling molecules, such as Wnt, Fgf, BMP, and Hh, and their signaling activity (Yan and Lin, 2009; Matsuo and Kimura-Yoshida, 2013; Mii and Takada, 2020). We showed that deletion of HS chains impairs local accumulation of Wnt3a and disrupts apical constriction. It is still unclear how Wnt ligands, trapped on HS chains, transduce their signals and regulate contraction of the actomyosin cytoskeleton only on the apical surface. An interesting future area of study is how locally accumulated Wnt proteins transmit their signals to local target cells.

Many studies have shown that Wnt ligands modulate cytoskeletal organization. It is well known that the β-catenin-independent pathway, or the non-canonical Wnt signaling pathway, mediates activation of Wnt ligands for cytoskeletal regulation without transcriptional activation (Montcouquiol et al., 2006; Schlessinger et al., 2009; Niehrs, 2012). In contrast, loss- or gain-of-function analysis of β-catenin in this study reveals that phosphorylation of MLC in RP cells is induced depending on β-catenin. Furthermore, MLC phosphorylation was also impaired in embryos defective in secretion of Wnt ligands from RP cells. These results strongly suggest that this phosphorylation is activated through the canonical Wnt/β-catenin pathway, in a transcription-dependent manner. Since ROCK and MLCK are involved in phosphorylation of MLC, it is plausible that these kinases or their modulators are regulated by Wnt/β-catenin signaling. In apical constriction of the neural plate during neural-tube closure in chick embryos, RhoA, which is activated by PDZ-RhoGEF guanine nucleotide exchange factor, activate ROCK and drives contraction of the actomyosin cytoskeleton (Nishimura et al., 2012). Thus, it seems probable that the Rho-mediated pathway is regulated by Wnt/β-catenin signaling. Identification of Wnt target gene(s) that induce phosphorylation of MLC is one of remaining challenges in understanding the mechanism of apical constriction mediated by Wnt/β-catenin signaling.

What is the significance of the Wnt-mediated morphological change of RP cells? RP cells dynamically change their morphology during neural development (Kondrychyn et al., 2013; Shinozuka and Takada, 2021). In the spinal cord region, the lumen gradually shrinks as the neural tube develops. In this reduction, apical tips of RP cells maintain contact with the dorsal side of the shrinking lumen, resulting in extension of RP cells along the midline. Production of Wnt proteins continues in extending RP cells and is required for proper change of RP cell morphology. In embryos in which Wnt secretion is specifically impaired in RP cells (*Wls* cKO embryos), RP cells are not aligned along the midline and the bundle of processes of RP cells frequently becomes branched (Shinozuka and Takada, 2021). In this study, we found that apical surfaces of RP cells are constricted by Wnt/β-catenin signaling, causing cell shape to become wedge-like at E10.5. Therefore, we propose that loss of this early constriction impairs subsequent RP cell elongation and bundle formation, ultimately leading to the apical surface expansion phenotype observed at later stages. Interestingly, in zebrafish embryos, disruption of the actin cytoskeleton, which is induced by inhibition of Zic6 or ROCK, impairs transformation of RP cells and reduction of the lumen (Kondrychyn et al., 2013). Cytoskeletal regulation by these molecules may generate mechanical force. It is possible that mechanical tension in RP cells caused by Wnt-induced cytoskeletal reorganization controls coordinated rearrangement of RP cells.

## Materials and methods

### Mice

This study was performed in accordance with Guidelines for Animal Experimentation of the National Institutes of Natural Sciences, with approval of the Institutional Animal Care and Use Committee of the National Institutes of Natural Sciences (#19A047, #20A062, #21A041). Every effort was made to minimize animal suffering during experimental procedures.

*Ext1 flox* mice were obtained from MMRRC (011699-UCD). *Ctnnb1 flox* (Brault et al., 2001)*, Ctnnb1 exon three flox* (Harada et al., 1999), *egfp-Wnt3a* KI (Shinozuka et al., 2019), *Wls flox* (Carpenter et al., 2010)*, Wnt1* KO (McMahon and Bradley, 1990), *Wnt1-Cre* (Danielian et al., 1998) and *Wnt3a* KO (Takada et al., 1994) mice have been previously described.

### Histology and immunohistochemistry

For hematoxylin and eosin (H&E) staining, embryos were fixed in 4% paraformaldehyde (PFA) over two nights at 4°C and then paraffin- or cryo-sectioned.

For immunohistochemistry, embryos were fixed in 3.5% PFA for 30 min at 4°C. Fixed tissues were embedded in O.C.T, compound (Sakura Finetek Japan) and cryosectioned at 14 µm. Immunohistochemistry was performed on cryosections of tissue as described below. Cryosections were incubated overnight at 4°C with the following primary antibodies: anti-β-catenin (sc-1496, Santacruz; 1:500), anti-Ezrin (ab4069, Abcam; 1:400), anti-GFP (598, MBL International; 1:500), anti-Myosin Heavy chain II-B (909901, BioLegend; 1: 400), anti-Phospho-Myosin Light Chain 2 (3674, Cell Signaling Technology; 1:400), anti-Gpr177 (ab176376, Abcam; 1:400) and anti-ZO1 (33-9100, Zymed; 1:500). Cryosections were then incubated overnight at 4°C with the following secondary antibodies at a 1:500 dilution: goat anti-mouse IgG Alexa Fluor 647 (A-21235, Invitrogen), goat anti-rabbit IgG Alexa Fluor 555 (A-21429, Invitrogen), donkey anti-mouse IgG Alexa Fluor 647 (A-31571, Invitrogen), donkey anti-rabbit IgG Alexa Fluor 488 (A-21206, Invitrogen) and donkey anti-goat IgG Alexa Fluor 555 (A-21432, Invitrogen). F-actin was stained with Alexa Fluor 555-phalloidin (A-34055, Molecular Probes). Tissue sections were counterstained with DAPI (Dojindo; 1:1000). Fluorescent images were acquired using an inverted confocal microscope (Nikon A1Rsi).

### Quantification and statistical analysis

Statistical analyses were performed using R software (version 4.1.3). Differences were assessed for statistical significance using Student's *t*-test. A *P* value of <0.05 was considered statistically significant. Box plots indicate the first and third quantiles and the median.

## Data Availability

The original contributions presented in the study are included in the article/supplementary material, further inquiries can be directed to the corresponding authors.
